# Kisspeptin signaling in the amygdala modulates reproductive hormone secretion

**DOI:** 10.1007/s00429-015-1024-9

**Published:** 2015-03-11

**Authors:** Alexander N. Comninos, Jelena Anastasovska, Meliz Sahuri-Arisoylu, Xiaofeng Li, Shengyun Li, Minghan Hu, Channa N. Jayasena, Mohammad A. Ghatei, Stephen R. Bloom, Paul M. Matthews, Kevin T. O’Byrne, Jimmy D. Bell, Waljit S. Dhillo

**Affiliations:** 1 Department of Investigative Medicine, Imperial College London, 6th Floor Commonwealth Building, Hammersmith Hospital, Du Cane Road, London, W12 0NN UK; 2 Metabolic and Molecular Imaging Group, MRC Clinical Science Centre, Imperial College London, Hammersmith Hospital, Du Cane Road, London, W12 0NN UK; 3 Division of Women’s Health, School of Medicine, King’s College London, Guy’s Campus, London, SE1 1UL UK; 4 Division of Brain Sciences, Imperial College London, Hammersmith Hospital, London, W12 0NN UK

**Keywords:** Amygdala, Hypothalamus, Reproductive axis, Manganese-enhanced MRI, Kisspeptin, Gonadotropin-releasing hormone neuron

## Abstract

Kisspeptin (encoded by *KISS1*) is a crucial activator of reproductive function. The role of kisspeptin has been studied extensively within the hypothalamus but little is known about its significance in other areas of the brain. *KISS1* and its cognate receptor are expressed in the amygdala, a key limbic brain structure with inhibitory projections to hypothalamic centers involved in gonadotropin secretion. We therefore hypothesized that kisspeptin has effects on neuronal activation and reproductive pathways beyond the hypothalamus and particularly within the amygdala. To test this, we mapped brain neuronal activity (using manganese-enhanced MRI) associated with peripheral kisspeptin administration in rodents. We also investigated functional relevance by measuring the gonadotropin response to direct intra-medial amygdala (MeA) administration of kisspeptin and kisspeptin antagonist. Peripheral kisspeptin administration resulted in a marked decrease in signal intensity in the amygdala compared to vehicle alone. This was associated with an increase in luteinizing hormone (LH) secretion. In addition, intra-MeA administration of kisspeptin resulted in increased LH secretion, while blocking endogenous kisspeptin signaling within the amygdala by administering intra-MeA kisspeptin antagonist decreased both LH secretion and LH pulse frequency. We provide evidence for the first time that neuronal activity within the amygdala is decreased by peripheral kisspeptin administration and that kisspeptin signaling within the amygdala contributes to the modulation of gonadotropin release and pulsatility. Our data suggest that kisspeptin is a ‘master regulator’ of reproductive physiology, integrating limbic circuits with the regulation of gonadotropin-releasing hormone neurons and reproductive hormone secretion.

## Introduction

Kisspeptin, encoded by the *KISS1* gene, is an arginine–phenylalanine amide neuropeptide that acts on the kisspeptin receptor (*KISS1R*). A critical role for the *KISS1*/*KISS1R* system in reproductive function was identified a decade ago from studies showing that humans with *KISS1R* or *KISS1* inactivating mutations failed to go through puberty (de Roux et al. 2003; Seminara et al. 2003; Topaloglu et al. 2012), while conversely activating mutations of *KISS1R* or *KISS1* resulted in central precocious (early) puberty (Teles et al. 2008; Silveira et al. 2010).

*KISS1* neurons in the hypothalamus secrete kisspeptin, which stimulates *KISS1R*-expressing gonadotropin-releasing hormone (GnRH) neurons (located predominantly in the preoptic area in rodents, POA) to release GnRH. GnRH then stimulates gonadotropin [luteinizing hormone (LH) and follicle stimulating hormone (FSH)] and subsequent sex steroid release from the anterior pituitary and gonads, respectively (reviewed in Pinilla et al. 2012). Key reproductive populations of *KISS1* neurons in rodents have been identified in two regions of the hypothalamus, the arcuate nucleus (ARC, equivalent to the infundibular nucleus in primates) and anteroventral periventricular nucleus (AVPV) (Clarkson et al. 2009).

However, *KISS1*/*KISS1R* expression is not limited to the hypothalamus (Clarkson et al. 2009; Lee et al. 1999), yet there is a paucity of data exploring the brain more widely. Given the established role of the *KISS1*/*KISS1R* system in reproduction, an area of particular interest is the amygdala, whose functions contribute to a broad range of social and reproductive behaviors (Murray 2007), as well as gonadotropin secretion and estrous cyclicity (Beltramino and Taleisnik 1978; Bagga et al. 1984). Furthermore, neuroanatomical studies have demonstrated neuronal projections between the amygdala and hypothalamic regions that regulate reproductive hormone release such as the ARC, AVPV and POA (Canteras et al. 1995; Hahn et al. 2003; Keshavarzi et al. 2014).

The finding of *KISS1R* expression within the human and rodent amygdala (Lee et al. 1999; Muir et al. 2001) as well as the more recent observation of sex steroid-dependent *KISS1* expression within the rodent amygdala (Kim et al. 2011) has heightened interest in the extra-hypothalamic roles of kisspeptin. We therefore hypothesized that kisspeptin has effects on neuronal activation and reproductive pathways beyond the hypothalamus and particularly within the amygdala.

**Fig. 1:**
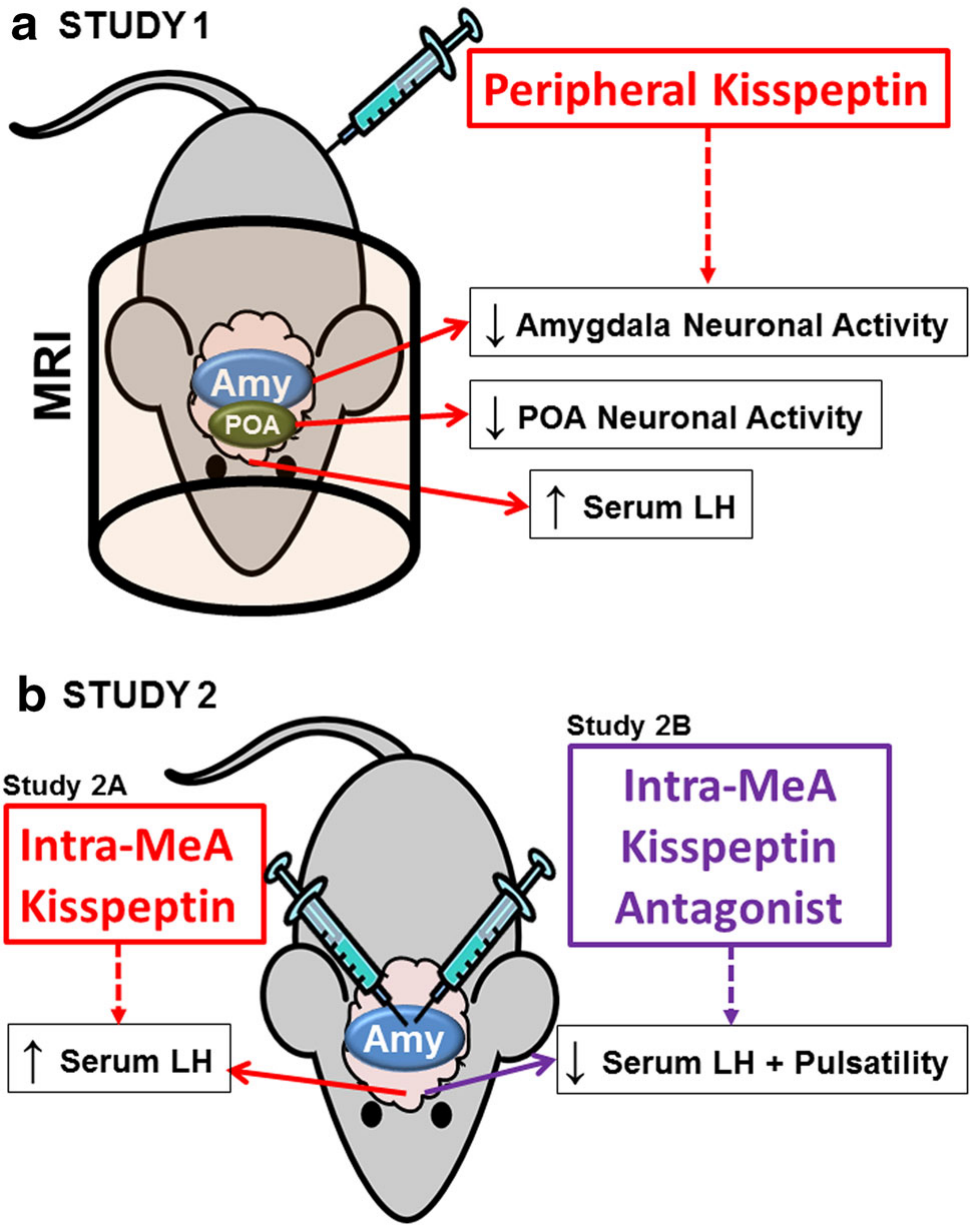
Scheduled summary of rodent studies. **a** Study 1: peripheral (ip) bolus of kisspeptin or vehicle was administered and subsequent changes in plasma kisspeptin, serum LH and neuronal activity in pre-selected CNS regions of interest (ROIs) were assessed (by manganese-enhanced MRI). Kisspeptin administration resulted in a significant increase in serum LH levels. This was accompanied by significant decreases in neuronal activity in the amygdala (Amy) and preoptic area (POA) after kisspeptin administration when compared to vehicle administration. **b** Study 2A: a bolus of kisspeptin or vehicle was administered directly into the medial amygdala (MeA) and subsequent serum LH and LH pulsatility was assessed. Administration of kisspeptin into the MeA increased LH secretion. Study 2B: a bolus of kisspeptin antagonist or vehicle was administered directly into the medial amygdala (MeA) and subsequent serum LH and LH pulsatility was assessed. Administration of kisspeptin antagonist into the MeA decreased serum LH and LH pulsatility

In a first test of our hypothesis (Study 1, Fig. 1a), we investigated the effects of peripheral kisspeptin administration on brain neuronal activity (including the amygdala) using manganese-enhanced MRI (MEMRI), as well as on LH secretion in rodents. We employed MEMRI, which utilizes the paramagnetic manganese ion as a contrast agent with ability to mimic Ca^2+^ and enter neurons through voltage-gated Ca^2+^ channels upon neuronal activation. This technique therefore permits temporal assessment of in vivo neuronal response to physiological and pharmacological stimuli (Kuo et al. 2007; Hankir et al. 2012).

In a second test of our hypothesis (Study 2, Fig. 1b), we assessed the effects of direct administration into the amygdala of kisspeptin or a kisspeptin antagonist on gonadotropin release to determine the functional significance of kisspeptin signaling specifically within the amygdala.

## Materials and methods

### Animals

#### Animal rights

Studies were performed in accordance with the United Kingdom Animals (Scientific Procedures) Act 1986 and were approved by the Imperial College London and King’s College London Ethical Review Committee.

Adult C57/BL6 male mice (8–10 weeks) and adult Sprague–Dawley female rats (12–14 weeks) obtained from Harlan Laboratories (Oxfordshire, UK) were housed under controlled conditions (12:12 h light/dark cycle, lights on 0700 hours; temperature, 22 ± 2 °C; food and water ad libitum). There were no weight differences between kisspeptin and vehicle-treated groups in each study.

### Study 1: effects of peripheral kisspeptin administration on plasma kisspeptin (Study 1A), serum LH (Study 1B) and CNS neuronal activity (Study 1C) in adult male mice (Fig. 1a)

We investigated the temporal effects of kisspeptin administration on circulating kisspeptin (Study 1A) and LH levels (Study 1B) during a 2-h time course to determine the optimal period for MEMRI scanning (Study 1C). Since the blood volume obtained from repeated tail clip sampling in mice is too small for multiple hormone analysis, two complementary studies were carried out (one for measurement of plasma kisspeptin (Study 1A) and the other for measurement of serum LH (Study 1B) following kisspeptin injection). Study 1B also served to confirm kisspeptin bioactivity.

#### Study 1A: effects of peripheral kisspeptin administration on plasma kisspeptin levels

Mice were exposed to equivalent conditions to MEMRI scanning but outside the scanner. Mice (*n* = 8/group) were anesthetized with 2 % isoflurane in oxygen at a flow rate of 2 L/min and maintained by 1 % isoflurane–oxygen. Blood (20 µl) was obtained from tail tip pre- (0 min) and post-bolus intraperitoneal injection (20, 40, 60, 120 min) of either kisspeptin-54 (0.04 nmol/g), or equivalent vehicle volume (gelofusine). Kisspeptin was dissolved in saline containing gelofusine (B. Braun Medical Ltd, UK) and was administered by intraperitoneal (ip) injection. This dose of kisspeptin was chosen as it has been previously shown to robustly increase LH secretion following ip injection in mice (Curtis et al. 2010; Jayasena et al. 2011).

Blood was collected in lithium–heparin cuvettes. Plasma was separated and stored at −20 °C. Measurement of plasma kisspeptin immunoreactivity (kisspeptin-IR) was performed using an established radioimmunoassay (Dhillo et al. 2005). Plasma kisspeptin levels were compared using two-way ANOVA. *P* < 0.05 was considered statistically significant.

#### Study 1B: effects of peripheral kisspeptin administration on serum LH levels

A separate but identical experiment to Study 1A was performed to determine the time profile of LH secretion following peripheral kisspeptin administration (*n* = 8/group). Blood was collected into plain cuvettes. Serum was separated and stored at −20 °C. Serum LH was assayed by magnetic-bead luminex immunoassay (inter- and intra-assay variation <20 and <15 %, respectively) (Milliplex Mouse LH Magnetic-Bead Assay, Merck Millipore, UK). Serum LH levels were compared using two-way ANOVA. *P* < 0.05 was considered statistically significant.

#### Study 1C: effects of peripheral kisspeptin administration on CNS neuronal activity using MEMRI

Having established a time course for the effects of peripheral kisspeptin administration on circulating kisspeptin and LH, we examined the effects of peripheral administration of kisspeptin on central neuronal activity during this time course in pre-determined 3D regions of interest (ROIs).

#### Protocol

Mice were acclimatized in a holding room adjacent to the MRI laboratory for 24 h pre-scanning. Mice were anesthetized as in Study 1A and 1B. MEMRI was performed by a 9.4-T MRI scanner (Agilent Technologies) with quadrature birdcage coil (Magnetic Resonance Labs) (Kuo et al. 2007; Hankir et al. 2012). Transverse slices covering whole brain were acquired repeatedly in an array, 66 times (127 min), using a 2D Fast Spin Echo multislice sequence with parameters: TR 1.8 s, effective TE 5.6 ms (6 echoes, spacing 5.6 ms, k-space center = 1), FOV 25 × 25 mm, matrix 192 × 192, 46 contiguous axial, 0.4-mm-thick slices and 2 averages. After the third acquisition, 100 mmol MnCl_2_ was infused by intravenous cannula (tail vein), at a rate of 0.2 ml/h and total dose of 0.5 μmol/g. Based on the results of Study 1, an effect of kisspeptin on circulating kisspeptin and LH levels is readily observed at 20 min post-kisspeptin administration. Manganese uptake reaches steady state within 20–60 min depending on brain region (Kuo et al. 2007); therefore, kisspeptin-54 (0.04 nmol/g, *n* = 8) or equivalent volume vehicle (*n* = 7) ip injection was administered simultaneously with initiation of MnCl_2_ infusion to ensure that the kisspeptin-induced changes in manganese uptake were detectable within the scanning period (Silva et al. 2004).

#### ROIs and image analysis

**Fig. 2:**
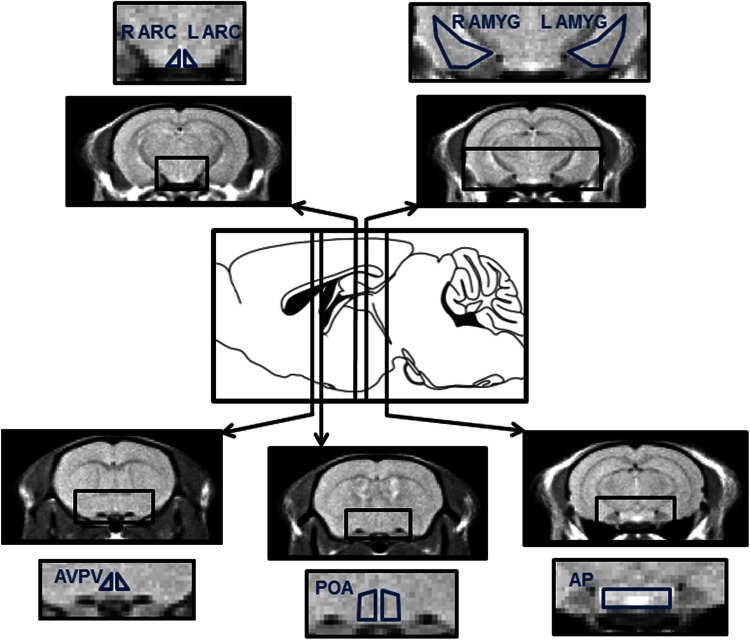
Adult mouse brain region of interests (ROIs) used for the MEMRI study. Relative positions of the transverse MRI slices within the adult mouse brain obtained during MEMRI scanning. Representative cross-sectional images are shown from MEMRI scans through 3D ROIs from which signal intensity profiles were generated corresponding to the amygdala, arcuate nucleus (ARC), anteroventral periventricular nucleus (AVPV), preoptic area (POA including medial septum), and anterior pituitary (AP)

Image-processing software (FSL, UK) was used to define ROIs on a standardized mouse brain template. 3D ROIs were neuroanatomically defined and corresponded to; left amygdala, right amygdala, left ARC, right ARC, AVPV, POA, and anterior pituitary with reference to standard mouse brain atlas (Paxinos and Franklin 2001) (Fig. 2). To ensure adequate manganese entry into the circulation only scans where signal intensity (SI) in the fourth ventricle increased >20 % over baseline within ten acquisitions were included. 4D brain images were extracted and spatially normalized (SPM5, FIL Methods Group; AFNI, Medical College of Wisconsin and FSL, UK), before SI time-course measurements were performed in the ROIs.

#### MEMRI data analysis

Data were normally distributed (assessed by Kolmogorov–Smirnov test). Percentage change SI from baseline (pre-manganese) to post-manganese infusion was calculated for each ROI (percentage enhancement, PE). PE was analyzed between kisspeptin and vehicle-treated groups by comparing entire time-course SI enhancement curve by general estimated equation (GEE) analysis as previously published (Kuo et al. 2007, 2010; Hankir et al. 2012). GEE performs general linear regression analysis using all values in each animal and therefore accounts for within-animal correlation between adjacent values (Zeger and Liang 1986). GEE analysis permitted assessment of significance of effect between kisspeptin and vehicle-treated groups. To assess quantitative size of effect, we utilized area under curve analysis (AUC). *P* < 0.05 was considered statistically significant.

### Study 2: effect of intra-medial amygdala (MeA) administration of kisspeptin (Study 2A) and kisspeptin antagonist (Study 2B) on LH secretion and LH pulsatility in adult female rats (Fig. 1b)

Study 1 showed that peripheral administration of kisspeptin inhibits neuronal activity in the amygdala and this is associated with an increase in LH secretion. In Study 2, we investigated the functional significance of kisspeptin signaling in the amygdala on LH secretion. To determine the effect of kisspeptin receptor agonism in the amygdala, we administered kisspeptin directly into the amygdala and measured LH release and pulsatility (Study 2A). To determine the role of endogenous kisspeptin signaling in the amygdala in LH release and pulsatility, we administered a kisspeptin antagonist directly into the amygdala (Study 2B).

#### Surgical procedures

All surgical procedures were carried out under anesthesia induced by ketamine (Vetalar, 100 mg/kg, intraperitoneal (ip); Pfizer, Sandwich, UK) and xylazine (Rompun, 10 mg/kg, ip; Bayer, Newbury, UK). Two weeks before experiments took place rats were bilaterally ovariectomized (OVX) and implanted with a silastic capsule (inner diameter 1.57 mm; outer diameter 3.18 mm; Sanitech, Havant, UK), filled to a length of 25 mm with 17 β-estradiol (E_2_; Sigma-Aldrich Ltd., Poole, UK) dissolved at a concentration of 20 µg/ml arachis oil (Sigma-Aldrich). The E_2_-containing capsule produced circulating concentrations of E_2_ within the range observed during the diestrous phase of the estrous cycle (Cagampang et al. 1991). At the time of ovariectomy, all rats were also fitted with a bilateral guide cannula (22 gauge; Plastics One, Roanoke, VA, USA) directed toward the MeA for microinfusion of pharmacological agents as previously described (Li et al. 2009). The stereotaxic coordinates for implantation being 3.4 mm lateral, 3.14 mm posterior to bregma, and 8.6 mm below the surface of the dura according to the rat brain atlas of Paxinos and Watson (1986). The guide cannula was secured using dental cement (Associated Dental Product, Swindon, UK) and fitted with a dummy cannula (Plastics One) to maintain patency. A stainless steel slotted screw (Instec Laboratories, Boulder, CO, USA) was affixed to the surface of the skull posterior to the guide cannula using dental cement. The rats were housed singly and allowed 10 days of recovery before being fitted with two indwelling cardiac catheters via the jugular veins, to facilitate serial blood sampling. The catheters were exteriorized at the back of the head and enclosed within a 30 cm metal spring tether (Instec Laboratories) secured to the slotted screw. The distal end of the tether was attached to a two-channel fluid swivel (Instec Laboratories), which allowed the rat free to move around the enclosure. Experimentation commenced 3 days later. Each animal was used once only. Correct cannula placement in the MeA was confirmed by microscopic inspection of 30 µm brain sections. Only data from animals with correct cannula placement were analyzed.

#### Study 2A: effect of intra-MeA kisspeptin administration on LH secretion

##### Protocol

On the morning of experimentation, an intra-MeA injection cannula (Plastics One, Roanoke, VA) with extension tubing preloaded with kisspeptin-10 or vehicle [artificial cerebrospinal fluid (aCSF)], was inserted into the MeA guide cannula. The distal end of the tubing, prefilled with aCSF was extended outside of the cage to allow remote microinfusion without disturbing the rat during the experiment. Microinfusion was performed manually over 5 min using a 5 µl syringe (Hamilton, Bonaduz, Switzerland). One of the two cardiac catheters was then attached via the fluid swivel to a computer-controlled automated blood sampling system, which allows for the intermittent withdrawal of small blood samples (25 µl) every 5 min for 6 h without disturbing the rats. Once connected, the animals were left undisturbed for 1 h before sampling commenced between 1000 and 1100 hours. After removal of each 25 µl blood sample, an equal volume of heparinized saline (50 U/ml normal saline; Wockhardt, Wrexham, UK) was automatically infused into the animal to maintain patency of the catheter and blood volume. After 2 h controlled blood sampling, kisspeptin-10 or vehicle was infused intra-MeA over 5 min. Rodents received a single dose of 100 pmol or 1 nmol kisspeptin-10 (*n* = 9 per treatment group) in 400 nl aCSF, bilaterally. These doses of kisspeptin were chosen as they have previously been shown to increase LH secretion following injection into the ARC or POA (Li et al. 2009). Control rats (*n* = 7) received 400 nl aCSF. Blood sampling continued for a further 4 h and samples were frozen in a −20 °C freezer until assay for LH. A double-antibody RIA supplied by the National Institute of Diabetes and Digestive and Kidney Diseases (NIDDK, Bethesda, MD, USA) was used to determine LH concentrations in the 25 µl whole-blood samples. Referenced preparation was rLH-RP-3. The sensitivity of the assay was 0.093 ng/ml. The intra-assay variation was 7.3 %, and the inter-assay variation was 10 %.

#### Study 2B: effect of intra-MeA kisspeptin antagonist administration on LH secretion and LH pulsatility

##### Protocol

For the infusion of a kisspeptin antagonist (peptide-234; Sigma-Aldrich, Dorset, UK) into the MeA, the intra-MeA injection cannulae preloaded with peptide-234 were set up in the same way as described above in Study 2A. Rats were administered 50 pmol peptide-234 (*n* = 9) in 400 nl aCSF over a period of 5 min after 2 h controlled blood sampling and then the same dosage was repeated on two further occasions at an interval of 30 min. These doses of kisspeptin antagonist were selected as they have been previously shown to inhibit LH pulsatility following injection into the ARC (Li et al. 2009). Blood sampling procedures were set up as described above for LH measurement. We selected the MeA in the amygdala as this is the predominant site for kisspeptin signaling within the amygdala (Kim et al. 2011).

#### LH pulsatility analysis

Detection of LH pulses was established by the algorithm ULTRA (Van Cauter 1988). The effect of pharmacological agents on pulsatile LH secretion was analyzed by comparing the mean LH pulse interval in the 2 h period preceding treatment with that in first hour and second to fourth hour post-treatment periods. LH pulse interval was calculated by the time interval between the peak value of each consecutive LH pulse detected in the appropriate analysis period. The LH pulse interval was not calculated for animals receiving intra-MeA injections of the larger dose of kisspeptin (1 nmol), since LH pulses were not reliably detectable during the post-treatment period because of the dynamic increase in circulating levels of LH. The significance of the effect of treatments on LH pulse intervals was compared with control animals injected with vehicle, at the same time points, as well as with the mean pulse interval during the 2 h pre-treatment period. The effect of pharmacological agents on overall LH secretion was calculated by comparing the area under the LH profile [area under the curve (AUC)] for the 2 h pre-treatment period with that in first hour and second to fourth hour post-treatment periods, using SigmaPlot version 11 (Systat Software, San Jose, CA, USA). Statistical significance was tested using one-way ANOVA followed by Dunnett’s test. All data were shown as mean ± SEM. *P* < 0.05 was considered statistically significant.

## Results

### Study 1A: peripheral kisspeptin administration increases circulating kisspeptin levels

**Fig. 3:**
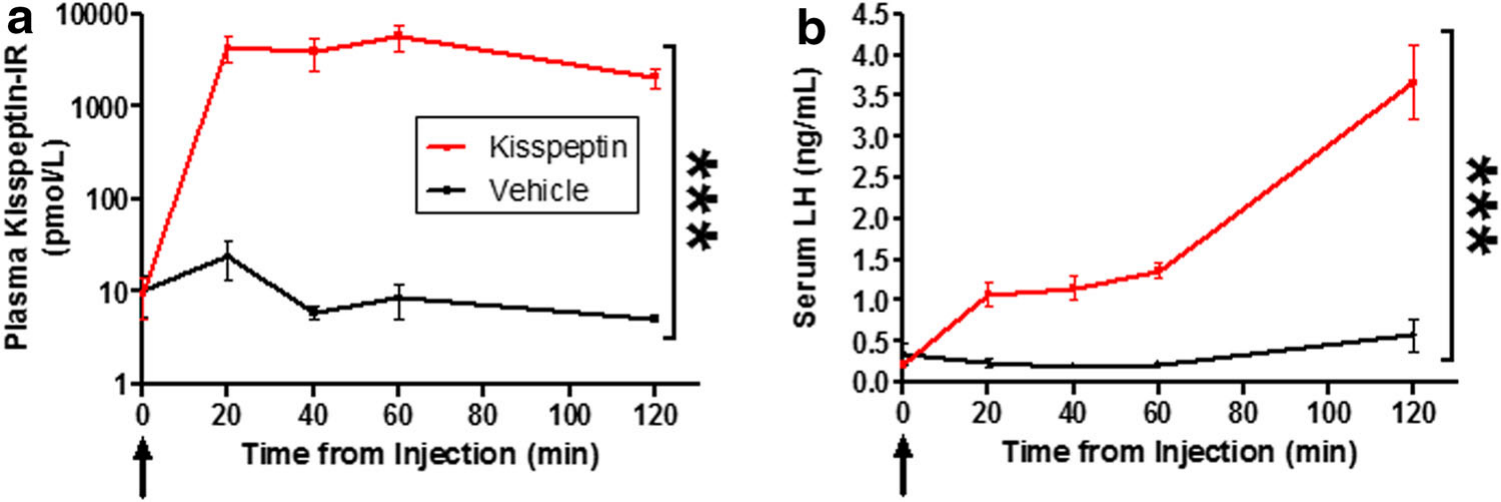
Effect of peripheral kisspeptin or vehicle administration on circulating kisspeptin and LH levels. Time course of increases in **a** plasma kisspeptin-IR and **b** serum luteinizing hormone (LH) after intraperitoneal injection of kisspeptin-54 (0.04 nmol/g) or vehicle at time 0 min in adult male mice. *n* = 8/group. ***P < 0.0001. Plasma kisspeptin-IR and serum LH increased significantly after kisspeptin compared to vehicle injection. Data presented as mean ± SEM. *Arrow* represents time of bolus injection of kisspeptin or vehicle

Vehicle administration had no effect on plasma kisspeptin-IR. Kisspeptin administration resulted in dramatic increases in plasma kisspeptin-IR compared to vehicle between 20 and 120 min post-administration (*P* < 0.0001, Fig. 3a).

### Study 1B: peripheral kisspeptin administration increases LH secretion

Vehicle administration alone had no effect on serum LH. However, kisspeptin administration increased LH compared to vehicle injection between 20 and 120 min post-administration (*P* < 0.0001) (Figs. 1a, 3b). The peak LH increase occurred at 120 min post-kisspeptin (vehicle +0.6 ± 0.2 ng/ml, kisspeptin +3.7 ± 0.5 ng/ml, *P* < 0.0001 vs. vehicle). The timings of the observed increases in LH (20–120 min) were similar to those at which increased plasma kisspeptin-IR was observed after kisspeptin administration (Fig. 3a).

### Study 1C: peripheral kisspeptin administration modulates CNS neuronal activity as determined by MEMRI

Having demonstrated increased LH secretion after kisspeptin administration, we proceeded to assess the effects of kisspeptin on central neuronal activity using MEMRI. We assessed the relative signal intensity (SI) as a marker of neuronal activation in selected, neuroanatomically defined 3D regions of interest (ROIs) over a 120-min time course (to encompass the hormonal changes observed in Study 1).

#### Amygdala

**Fig. 4:**
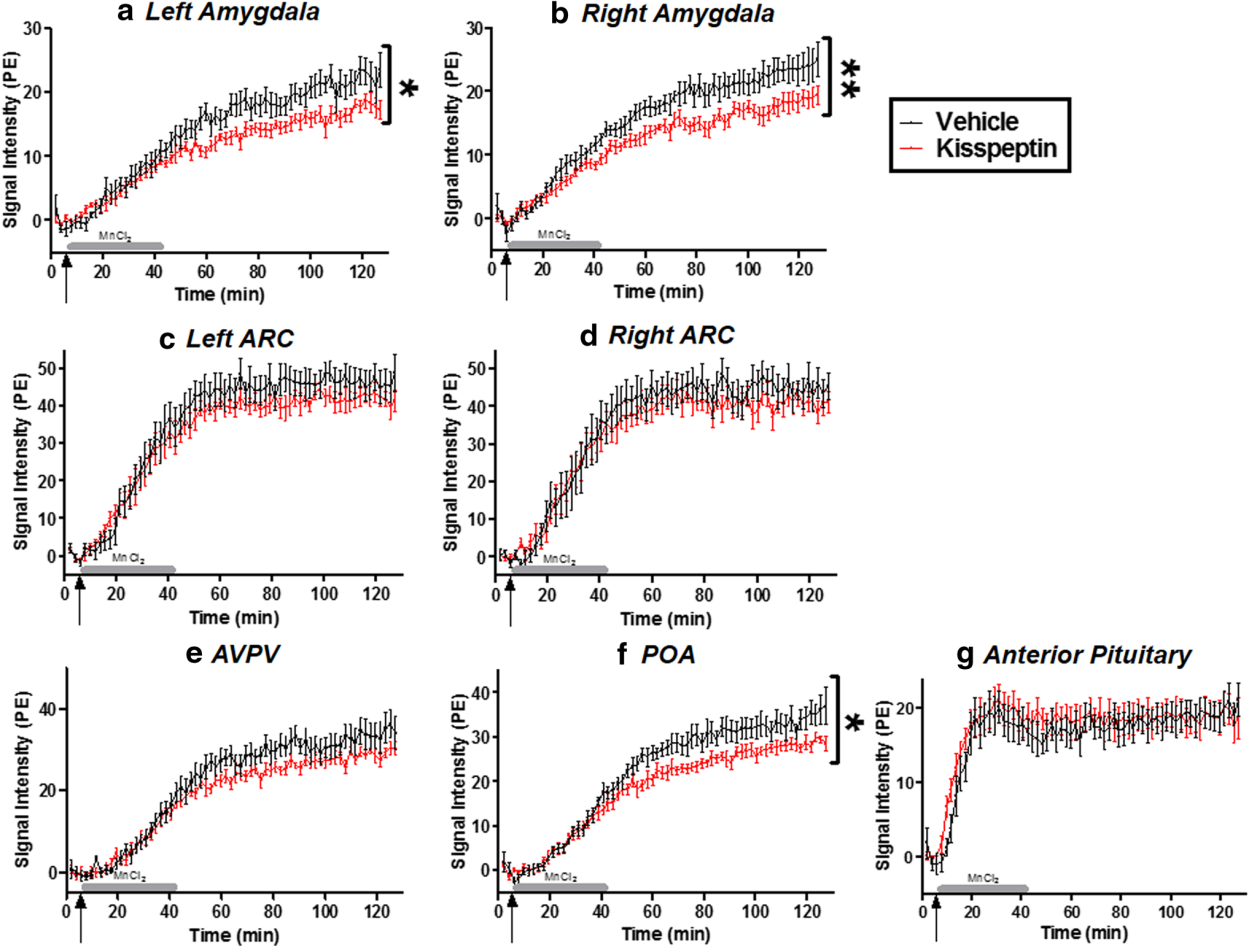
Changes in CNS neuronal activity following kisspeptin or saline administration. Time course of T1-weighted MRI signal change in the left amygdala (**a**), right amygdala (**b**), left ARC (**c**), right ARC (**d**), AVPV (**e**), POA (**f**) and anterior pituitary (**g**) after intravenous MnCl_2_ infusion into adult male mice also receiving an intraperitoneal injection of kisspeptin (*n* = 8), or vehicle (*n* = 7). *Arrows* indicate the start time of MnCl_2_ infusion and bolus injection of kisspeptin or vehicle. *Gray bar* represents duration of MnCl_2_ infusion. Signal intensity (SI) was measured as percentage enhancement (PE) over baseline. **P < 0.01, *P < 0.05. Data presented as mean ± SEM

Reduced SI was observed in the amygdala after kisspeptin administration compared to vehicle alone (GEE analysis: vehicle left amygdala vs. kisspeptin left amygdala, *P* = 0.0257; vehicle right amygdala vs. kisspeptin right amygdala, *P* = 0.0085, Figs. 1a, 4a, b).

The mean SI decreased by 20 % in the left amygdala and 22 % in the right amygdala (mean SI AUC in PE.min, vehicle left amygdala 885.1 ± 82.2, kisspeptin left amygdala 704.1 ± 40.3; vehicle right amygdala 983.4 ± 76.6, kisspeptin right amygdala 762.3 ± 51.9). This reduction of SI in the amygdala (Fig. 4a, b) occurred at a similar time to the increases in circulating kisspeptin (Fig. 3a) and LH (Fig. 3b).

#### ARC and AVPV

No significant differences in SI between kisspeptin and vehicle administration were observed in either the ARC (Fig. 4c, d) or AVPV (Fig. 4e).

#### POA

Reduced SI was observed in the POA after kisspeptin administration compared to vehicle (*P* = 0.0234, Figs. 1a, 4f).

#### Anterior pituitary

Although there was a trend towards an initial increase in SI in the first 20 min after kisspeptin injection, no significant differences were observed over the full time course of the experiment (Fig. 4g).

### Study 2A: intra-medial amygdala (MeA) administration of kisspeptin stimulates overall LH secretion

**Fig. 5:**
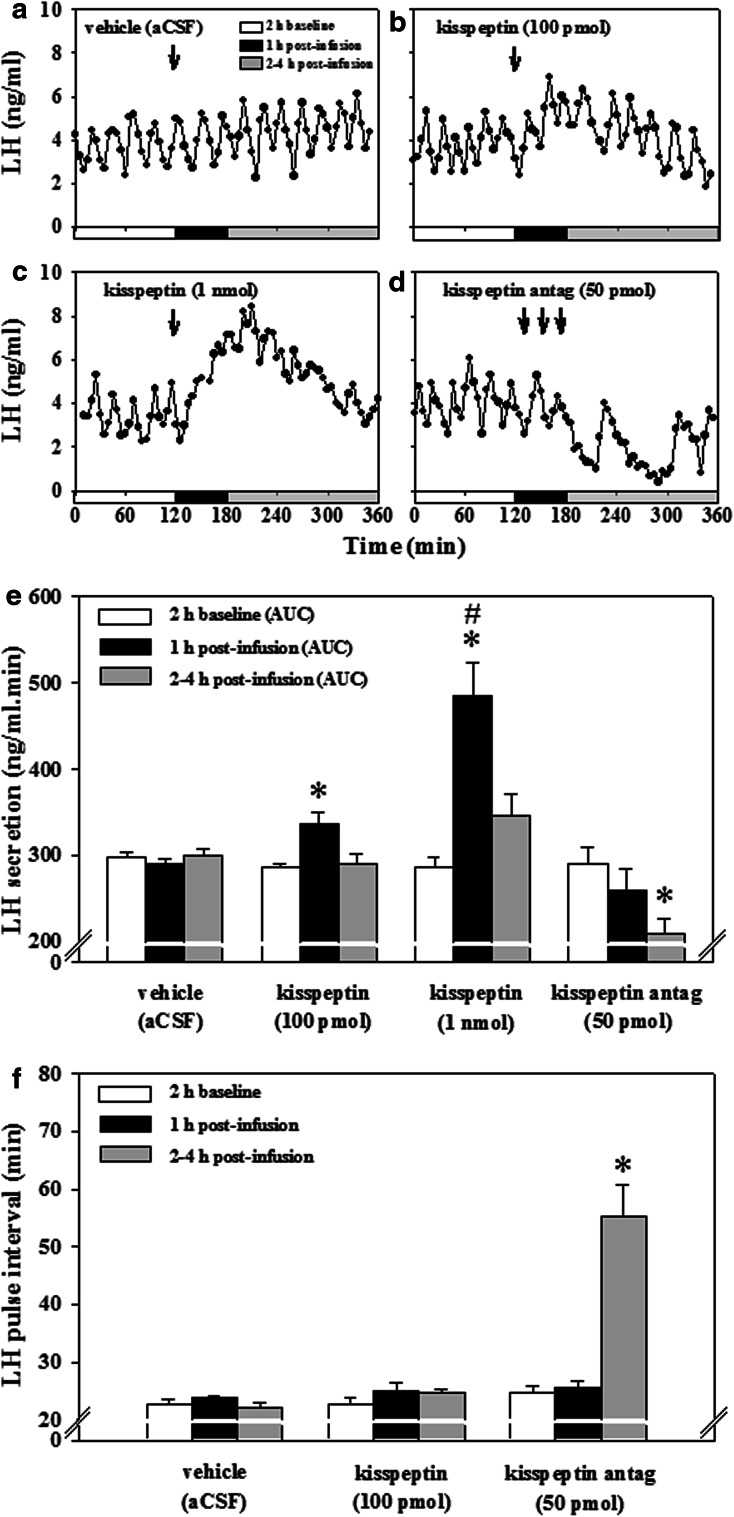
Effect of kisspeptin or kisspeptin antagonist injection into the amygdala on LH secretion. Effect of micro-injection of kisspeptin or kisspeptin antagonist (peptide-234) into the medial amygdala (MeA) on LH secretion and pulse frequency in ovariectomized rats implanted with estradiol capsules. Representative LH profiles demonstrating the effect of intra-MeA administration (at time marked by ‘*down arrow*’) of vehicle (**a**), kisspeptin 100 pmol (**b**), kisspeptin 1 nmol (**c**), or kisspeptin antagonist (**d**) on pulsatile LH secretion. Direct administration of kisspeptin into the amygdala dose-dependently increased LH secretion (**b**, **c**) shown by increased area under curve (AUC) of the LH profile (**e**). Direct administration of kisspeptin antagonist into the amygdala decreased LH AUC (**e**) and more specifically suppressed LH pulse frequency (**d**, **f**). The prolonged increase in LH in response following 1 nmol kisspeptin administration precluded reliable LH pulse determination. *P < 0.05 vs. vehicle; ^#^ *P* < 0.05 vs. dose of 100 pmol kisspeptin at same time point; *n* = 5–7 per group. Data presented as mean ± SEM

Having observed decreased neuronal activity in the amygdala following peripheral kisspeptin administration, we proceeded to determine the role of kisspeptin signaling in the amygdala in LH secretion. To do this, we administered kisspeptin directly into the amygdala. Direct intra-MeA administration of kisspeptin resulted in a dose-dependent increase in circulating levels of LH determined by AUC (Figs. 1b, 5b, c, e; *P* = 0.007, kisspeptin vs. vehicle 100 pmol; *P* = 0.002, kisspeptin 1 nmol vs. vehicle; *P* = 0.002, kisspeptin 1 nmol vs. kisspeptin 100 pmol). LH pulse frequency was not affected by intra-MeA injection of 100 pmol kisspeptin (Fig. 5f), and the dramatic and prolonged increase in LH in response to 1 nmol kisspeptin precluded reliable LH pulse detection (Fig. 5c, e, f). Intra-MeA administration of vehicle (aCSF) did not affect AUC or LH pulse frequency (Fig. 5a, e, f).

### Study 2B: intra-MeA administration of a kisspeptin antagonist decreases pulsatile and overall LH secretion

To determine if endogenous kisspeptin signaling in the amygdala affects LH secretion, we administered kisspeptin antagonist directly into the amygdala. Intra-MeA administration of the kisspeptin antagonist, peptide-234, decreased the frequency of LH pulses (i.e. increased pulse interval Figs. 1b, 5d, f; *P* < 0.001, vehicle vs. kisspeptin antagonist) and reduced LH secretion (Figs. 1b, 5e; *P* = 0.041, vehicle vs. kisspeptin antagonist).

## Discussion

Although our understanding of the *KISS1*/*KISS1R* system has improved dramatically in the past decade, the vast majority of work has focussed on the hypothalamus. The effects of kisspeptin in other areas of the brain have been less well characterized. Additional data are now required as we refine our understanding of kisspeptin action and develop therapeutics based on the *KISS1*/*KISS1R* system. To this end, recent advances in neuroimaging afford us a unique opportunity to study kisspeptin further.

Current evidence suggests that the amygdala exerts a predominantly ‘inhibitory brake’ on the reproductive axis and on reproductive behavior. For example, electrical stimulation of the amygdala delays puberty (Bar-Sela and Critchlow 1966). Conversely, lesioning of the amygdala results in hypersexuality (Kluver and Bucy 1997), increased circulating LH (Lawton and Sawyer 1970), and prevents the stress-induced suppression of LH pulses (Lin et al. 2011). Here, we report for the first time that, in adult rodents, peripherally administered kisspeptin inhibits neuronal activation in the amygdala and is temporally associated with an increase in LH secretion (Fig. 1a). Therefore, these data suggest that kisspeptin administration may release the tonic ‘inhibitory brake’ exerted by the amygdala on the reproductive axis to stimulate gonadotropin secretion.

Although *KISS1* and *KISS1R* are expressed in the rodent (and human) amygdala (Lee et al. 1999; Muir et al. 2001; Clarkson et al. 2009; Kim et al. 2011), peripherally administered kisspeptins are not thought to cross the blood–brain barrier (Herde et al. 2011). Previous studies have shown that, although *central* administration of kisspeptin increases *C*-*FOS* expression in GnRH neurons in the POA (Irwig et al. 2004), no change in GnRH neuronal *C*-*FOS* expression has been observed after *peripheral* kisspeptin administration in adult mice (d’Anglemont de Tassigny et al. 2010). This is in keeping with both our current study and previous studies suggesting that peripherally administered kisspeptin may predominantly act on GnRH neuron dendritic terminals outside the blood–brain barrier which therefore do not undergo cell body activation (Herde et al. 2011; Xu et al. 2012).

However, it has previously been shown that *peripheral* kisspeptin administration can alter the neuronal activity of *central* neurons (Scott and Brown 2011). In the current study, we observed an overall inhibitory effect of peripherally administered kisspeptin on neuronal activity in the amygdala in association with an increase in LH secretion. Similarly, we observed a decrease in neuronal activity in the POA in association with an increase in LH secretion (Fig. 1a). It is important to consider that MEMRI cannot distinguish directly between activation of one population of neurons and deactivation of another within the same ROI, and hence represents an overall net effect.

It is interesting to consider reasons for the decrease in net neuronal activity observed in the amygdala and POA following peripheral administration of kisspeptin. It is well established that kisspeptin can modulate central γ-aminobutyricacidergic (GABA) activity (Zhang et al. 2009; Neal-Perry et al. 2009). Neal-Perry et al. (2009) using microdialysis techniques in adult rodents in vivo, observed that kisspeptin administration directly into the POA decreased local GABA release by over 50 %, while simultaneously stimulating robust LH secretion. Consistent with this Zhang et al. (2009), demonstrated that kisspeptin administration decreases the GABAergic input to GnRH neurons in the POA in adult rodent brain slices. Furthermore, GABA neurons inhibit GnRH activity and resultant LH secretion (Neal-Perry et al. 2009; Garcia-Galiano et al. 2012; Zhang et al. 2009; Zuure et al. 2013; Martin et al. 2014). Therefore, it is possible that the decreased neuronal activity detected in the amygdala and POA in the current study represents a decrease in GABA neuronal activity as a consequence of kisspeptin administration. A reduction in GABA activity would lead to reduced inhibition of GnRH neurons and hence an increase in LH secretion as observed in the current study. In keeping with this, there are established networks of kisspeptin and GABA neurons and interneurons in both the amygdala and POA with projections between these areas (Canteras et al. 1995; Veinante et al. 1997; Hahn et al. 2003; Pinilla et al. 2012; Di Giorgio et al. 2014; Keshavarzi et al. 2014).

An alternative hypothesis to explain the decreased neuronal activity observed in the POA is provided by the nitric oxide (NO) synthesizing neuronal population within the POA which has been implicated directly in the regulation of GnRH neuronal activity in the mouse (Clasadonte et al. 2008) and rat (Grossman et al. 1994). These neurons are able to sense circulating kisspeptin and relay this information onto adjacent GnRH neurons (Hanchate et al. 2012), the activity of which is decreased by neuronal NO in keeping with the current study (Clasadonte et al. 2008). Recent mathematical modeling suggests that the neuroendocrine brain could use these peripheral hormone-mediated episodes of NO release to synchronize GnRH neuronal activity resulting in increased gonadotropin release consistent with the LH increases observed in our study (Bellefontaine et al. 2014).

We also examined manganese uptake in other hypothalamic regions with well-established reproductive functions. Neuronal activity in the ARC and AVPV was unaffected by peripheral kisspeptin administration compared to vehicle. This is consistent with th absence of the expression of *KISS1R* in these areas in mice and the inability of peripherally administered kisspeptin to reach these areas (Herbison et al. 2010).

Finally in Study 1, we evaluated the influence of kisspeptin on manganese uptake within the anterior pituitary, the site of LH release from the gonadotropin response to GnRH. Although we observed an initial non-significant increase over the first 20 min, this did not continue through the full time course. This could be due to rapid habituation of the gonadotroph cell population by the pharmacological concentrations of kisspeptin administered which are known to cause sustained GnRH release which stimulates the gonadotrophs (Han et al. 2005). However, given that gonadotrophs represent a fraction of the cell population of the anterior pituitary, it is also possible that any effect on gonadotroph activity was masked by other, non-gonadotroph activity.

An important consideration of the MEMRI technique in Study 1 is the use of anesthesia which typically suppresses neuronal activity and hence potentially makes any changes in response to an external stimulus harder to detect. However, a degree of anesthesia is required to keep the animal stationary and minimize any effects of stress on the reproductive axis (Chand and Lovejoy 2011). In addition, the depth of anesthesia correlates with suppression of MEMRI signal intensity (Silva and Bock 2008). Hence the anesthetic dose used in this study was based on previous studies in our laboratory which ascertained the lowest anesthetic dose at which reliable immobility was achieved while permitting assessment of the effects of peripherally administered hormones on CNS neuronal activity (Chaudhri et al. 2006; Kuo et al. 2006, 2007). The effects of the anesthesia should not significantly limit the results of the current study as both vehicle- and kisspeptin-treated mice received the same anesthetic protocol. Furthermore, the LH increases observed following kisspeptin administration in the current study were similar to those seen following peripheral administration of kisspeptin to non-anesthetized rodents in other studies suggesting that kisspeptin action is indeed maintained under anesthesia (Curtis et al. 2010; Jayasena et al. 2011).

Having observed in Study 1 that peripheral kisspeptin administration results in decreased neuronal activity in the amygdala and is associated with increased circulating LH levels (Fig. 1a), we next investigated the role of kisspeptin signaling within the amygdala in LH secretion and LH pulsatility in Study 2 (Fig. 1b). To do this, we used a well-established rat model (Li et al. 2009) to inject peptides directly into the amygdala and sampled blood frequently to evaluate LH secretion and pulsatility in freely moving animals. This is technically difficult to perform in mice. To determine the role of amygdala *KISS1R* agonism in LH secretion, we infused kisspeptin directly into the amygdala. We observed that intra-amygdala infusion of kisspeptin stimulated LH secretion. To test for a role of endogenous amygdala kisspeptin in LH release, we infused a kisspeptin antagonist directly into the amygdala. This resulted in a decrease in LH secretion and pulsatility (Fig. 1b).

Therefore, these data suggest that endogenous amygdala kisspeptin signaling regulates not only GnRH secretion (and hence LH secretion), but also may regulate the GnRH pulse generator per se to alter LH pulsatility. Furthermore, previous work has also demonstrated an increase in LH secretion after intra-POA administration of kisspeptin highlighting the importance of both the amygdala and POA in stimulating LH secretion via kisspeptin signaling (Li et al. 2009). This, in addition to our finding of marked changes in neuronal activation within the amygdala and POA following peripheral kisspeptin administration, provides evidence of a novel pathway involving the amygdala and hypothalamus through which kisspeptin mediates its neurophysiological effects.

It is important to note that we performed Study 1 in male and Study 2 in female adult rodents. Male mice were used in Study 1 as they do not have cyclical changes in reproductive hormones and *KISS1* expression (Kim et al. 2011) coupled with our experience in employing MEMRI in mice (Kuo et al. 2006, 2007, 2010; Hankir et al. 2012). To allow for accurate intra-amygdala kisspeptin injection and sensitive LH pulse detection we employed a female rat model (OVX + E2) established in our laboratory for Study 2 (Li et al. 2009; Lin et al. 2011). Importantly, *KISS1* expression in the amygdala is similar in both male and female mice and rats (Kim et al. 2011).

An additional consideration is the possibility of diffusion of administered kisspeptin from the amygdala into the ventricular system of the rats, resulting in an effect via the hypothalamus to trigger LH secretion. The dose of kisspeptin which stimulated LH secretion when administered directly into the medial amygdala in rats in the current study was 100 pmol. However, previous data have demonstrated that the intracerebroventricular (icv) administration of 100 or 300 pmol doses of kisspeptin in rats does not significantly stimulate LH secretion when compared to vehicle (Thompson et al. 2004; Pheng et al. 2009). This suggests that in our current study, kisspeptin is having its actions within the amygdala rather than diffusing into the ventricular system to have effects at the hypothalamus. However, local genetic manipulation studies would add further confirmation of a direct action of kisspeptin in the amygdala.

Our findings open up future directions in which roles of kisspeptin outside the hypothalamus may be studied further using neuroimaging techniques. Given our observations of changes in amygdala neuronal activity in response to kisspeptin and a direct association with LH secretion, future work may focus on the *KISS1*/*KISS1R* system and sexual behaviors attributed to the amygdala. Along these lines, Kauffman and colleagues (2007) demonstrated that *KISS1R* expression is essential for olfactory-mediated partner preference behavior but further behavioral studies are now warranted.

In summary, we demonstrate that peripheral kisspeptin administration decreases overall neuronal activity within the amygdala and increases LH secretion (Fig. 1a). An increase in LH secretion is also observed when kisspeptin is administered directly into the amygdala, while conversely blocking of endogenous kisspeptin signaling within the amygdala inhibits LH secretion and LH pulsatility (Fig. 1b).

To our knowledge, this is the first demonstration of kisspeptin signaling outside the hypothalamus directly modulating reproductive hormone release. Our data therefore provides evidence that kisspeptin is a ‘master regulator’ of reproduction, integrating limbic circuits with the modulation of GnRH neurons and gonadotropin release.
